# Botany, in Its Relations with Medicine—No. 2

**Published:** 1877-11

**Authors:** W. T. Grant

**Affiliations:** Atlanta, Ga.


### BOTANY, IN ITS RELATIONS WITH MEDICINE—No. 2.

BY W. T. GRANT, M.D., ATLANTA, GA.

It must now be admitted that the remedies which are furnished to the physician by chemistry are quite as valuable as those which are derived from the vegetable kingdom. I mean, when I speak of the chemical medicines, those which are derived from inorganic nature by the chemist's art. Even admitting this much, the superiority must be awarded to botany, for the reason that, while the really excellent inorganic medicines may be counted on the fingers, the others can only be numbered by the hundreds; and this, too, in face of the fact that the study of chemistry has of late years received an extraordinary impetus, while botany has been entirely ignored.

It seems to me that, while neither of these sciences should be tabooed, that they should go hand in hand ; and I cannot but believe that, together, they will constitute a power, or resource, to which the physician may confidently appeal, in any extremity. The harmonious working of the two sciences, and their great results, are shown in the fact that botany pointed out the cinchona bark, and chemistry extracted the quinia and other alkaloids for the convenient use of the physician. Botany presented the poppy, and chemistry took the inspissated juice (opium) and gave us the morphia from it. They are thus complementary, the one to the other ; and the science of medicine can never be complete until they shall both receive a full share of the physician's attention.

Of the two sciences, botany is far the easiest to learn, and involves literally no expense beyond the purchase of less than half a dozen books. It would be the height of absurdity to expect all the students who graduate in medicine to be competent chemists. If they comprehend the very general principles of the science, it will be as much as is expected of them. But with botany the case is very different. No preparation is needed, no expensive apparatus to buy—nothing is to be done but study and use common sense. In botany, the objects of study are all around us, requiring little labor to gather, and very little to understand. So that it is entirely true, that there is no comparison whatever between the two sciences in ease of acquisition or in convenience of material. Botany is easy to acquire, and simple ; chemistry difficult and complicated. The objects of botany are at every man's door, while the materials for chemical studies are expensive and sometimes difficult to procure.

Reasoning from such a comparison, the conclusion is easy, that while it is no difficult matter for every student of medicine to become a good botanist, exceedingly few ever can or will become good chemists. And it seems to me that this is only consistent with the "eternal fitness of things," as there is no need that all *should* be chemists, a few good chemists being entirely sufficient; whereas, it is not only important, but very essential, that *every* physician should be a good botanist. Only in this way can we have a sufficient number of properly qualified observers in the field studying our flora.

I do not wish it understood that I would disparage the study of chemistry in the least particular. Such is far from my intention; it is a valuable auxiliary to the physician in his combat with dire disease, and I render to it every credit to which it is entitled. I have instituted these comparisons only to show that the profession only stultifies itself, and rejects one of the most invaluable aids as long as botany is ignored. I would not banish chemisty from the curriculum of our medical schools, but I would introduce botany there, so well satisfied am I that it would repay tenfold value for the attention bestowed upon it, and add quite as much to medicine as any other one in the family of the medical sciences.

Botany has been too long ignored by the regular profession, and left in the hands of charlatans. And it has now become essential that it should be taught in every medical college, if we would have materia medica and therapeutics advance equally with the other branches of medicine. And that college which shall first establish a chair of botany will not only receive but deserve the plaudits of the future. Independently of all this, the value of a course of medical lectures which includes in its course a well taught series upon botany will attract to itself and retain large numbers of students who have the ability to recognize the fact that no medical course can possibly be complete which does not embrace botany in its curriculum. And every first course student will make that institution his *alma mater*, because he cannot acquire similar advantages elsewhere.

I know of no medical institution anywhere, not even those which claim to be botanic par excellence, that teaches botany. If there be such an one, I know nothing of it. I have never yet met a graduate of any institution whatever who had any knowledge of practical botany.

As is said in another part of this essay, we have excellent physicians and some very good botanists, but it is essential that the two should be combined in one man before medicine can ever expect to reap the rich harvest of remedies which is ripe for the sickle. This necessary combination is very like the combination which is absolutely necessary in the great astronomical discoverers. There he must unite the astronomer proper with the mathematician, before he can expect to make any grand discovery. So in this case, the physician must also be a botanist before he is qualified to take hold of, and deal with any of the great problems which may be found within the province of materia medica and therapeutics.

Independently of the great results which must necessarily accrue to our profession so soon as every physician shall become a botanist, it seems to me that it would be a great pleasure and satisfaction to the practitioner to possess a competent knowledge of this subject.

I have no doubt but that many valuable discoveries of remedies have been suppressed, just because the physician did not know the proper name of the plant he may have used in the particular case. I once read an article by a competent physician, in which he recommended a strong tea of the "cow-nettle-balls," in puerperal convulsions, as a very reliable remedy. Probably very few readers of his paper at a distance would know what he meant. But if he had said that he had used the ripe fruit of solanum carolinianum then a reader in Europe could at once have put his finger upon the plant anywhere it might grow.

